# The Latest Sphæro Bacterium—A Good Thing to Have in the Family

**Published:** 1882-11

**Authors:** 


### Editorial.

---

### THE LATEST SPHÆRO-BACTERIUM.—A GOOD THING TO HAVE IN THE FAMILY.

We publish in this number two original communications, in which will be found a record of certain extraordinary facts, relating to a case of tetanus.

The circumstances of this case may be briefly recapitulated, as follows: Two eminent physicians of this city are in attendance upon a lad affected with perfectly typical tetanus, resulting from a wound in his hand inflicted by the explosion of a toy pistol. They determine to examine his blood with the microscope, and, having done this, discover that it is swarming with organisms precisely similar to the sphæro-bacteria recognized in the ill-conditioned pus removed from an abscess communicating with a joint. The blood of a horse affected with tetanus is then placed by them under the same objectives, and in it also similar organisms are discovered. Not content with pushing their investigations to this point, they proceed to examine the mother of the boy who is their patient, and also another of her children; and to their surprise find in the blood of each an abundance of the same germs. A neighboring frog-pond is finally searched, and found to furnish masses of the same bacteria. The boy recovers from his tetanus, and is taken before a body of scientific gentlemen in this city, where his blood, still swarming with bacteria, is placed on slides and examined by a dozen or more physicians and others of unquestioned discrimination and veracity. All are able to recognize the existence of the bodies to which their attention is directed.

These are, indeed, remarkable facts which do not precisely correspond with the lessons we have been taught of late in the rapidly multiplying inferences deduced from the studies of the germ-theorists. What, then, let us ask, is their real significance, and how shall they be wisely interpreted?

There could scarcely be a more appropriate time than this in which to ask our friends, the micro-pathologists, to withdraw their keen eyes for a moment from their mounted specimens, and to indulge in what Mr. Tyndall was pleased to recommend as the scientific use of the imagination. Here, surely, the theoretic philosopher should be able to step in, and, reviewing the entire field, evolve from the mass of accumulated facts an hypothesis which will at once and satisfactorily explain them all, and gratify the scientific appetite which they have naturally stimulated in the highest degree.

For example, there is the hypothesis which might be termed, following the clever suggestion of Max Müller, the "Too-too" theory. According to this, the sphæro-bacterium, of the blood at least, should be recognized as an altogether pleasant and lovely schizomycete, dancing away its dizzy life in the vascular channels with a levity born of utter guilelessness. Its ultra development at either extremity, suggests a species of bi-polar assimilation, by which at either end it is capable of making part of itself rod-bacteria, for example, and other small game found poaching on its preserves. Compared with such an aliment, how the famous Diet of Worms shrinks into utter insignificance! This, doubtless, is the meat on which our micrococcus hath fed, that it hath grown so great! In this simple way, also, we discover an explanation of its sprightliness; for who does not know that only well-fed and well-housed organisms are capable of spinning in such lively motion, or, coupled with their fellows in chains of spores circling around the red blood disks, of engaging in the pastimes peculiar to youthful and healthful activity? Here, indeed, is no spectral corpuscle like that invisible and ghostly hæmatoblast over which Norris and Bizzozero have set themselves in array to do battle. Here, neither, is a coy and reluctant bacillus which refuses to materialize save under the persuasive influence of Weigert's fuchsine, or of Hansen's methyl-violet, or of Gibbes' diphenolrosanilin. This is a robust, rotund and almost portly micrococcus, with either a double chin or a double belly, as the observer may choose to regard it, and an expression of benevolence in its every feature, and of happiness in every one of its motions.

Or, again, we are at liberty to adopt an explanation, which, still following Max Müller, we might term, the "Gone wrong" theory. This is, we may remark in passing, dimly shadowed forth in the remarks made lately by Mr. Faraday, in connection with the subject of bacilli, before the British Association for the Advancement of Science. According to the latter, there may be several varieties of these interesting organisms, which outwardly resemble each other in every feature, but exhibit the widest divergence in their naughty actions. Thus there are some which are as gentle as any sucking dove, and as harmless as a kitten, a class to which may be assigned the Curtis-Brower sphæro-bacterium. These have, however, half-brothers, not even to be distinguished from the former by a strawberry mark on the sinister sphere of the dumb-bell, who have gone utterly wrong, and thus become, by a process of natural cultivation "in the presence of a reduced amount of oxygen," half-starved vagrants. These are they who have "acquired a parasitic habit," or, being slightly better fed and housed than the very lowest of these criminal classes, have undergone "transformation from a harmless saprophyte" into a thoroughly vicious "sport." This view is so entirely natural and in accord with the doctrines of evolution that it commends itself at once to the student of social morphology. If it could but once achieve a general acceptance, there is probably not a lover of the human race in the whole world, who would not regard with feelings of unspeakable horror, the proposition of M. DeKorab, who, finding lately that helenine exercises a destructive action upon these germs, proposes to engage aniline, red-handed, in the task of obliterating, in one common slaughter, both varieties, the micrococci of good and the micrococci of evil, examples of which are well furnished in the spæro-bacterium of Messrs. Curtis and Brower on the one hand, and the tubercle bacillus, on the other.

But a third, and decidedly the most plausible explanation of all these facts, might be described as the "Saw-dust Doll" theory.

According to the latter, all these micrococci should be regarded in the light of inestimable benefactors of the human family. It is only when men, women and children begin to lose their precious bacteria, that danger arises. Thus when the sputa, the pus, or physiological secretions become carriers of these natural blood-purifiers out of the body, it is time to take counsel of the wise, not to commit the fatal blunder of engaging in an attempt to still further reduce the number and efficacy of the bacteria which remain. In this view, a tardy justice is done to these small friends of the family of man, whom we had begun to regard with a hatred and malevolence for which Ehrlich, Koch, Eklund, Neisser, Carter, and we know not how many more, should be held to a rigid accountability. When, by a yet undescribed pathological process of weakening of the vascular walls, the sphæro-bacteria, which should rather have remained within to perform their kindly offices, have leaked out, whether into the outer world or into other tissues of the human frame, there have we discovered them, and there, too, have we blindly misconstrued their presence. Starved and attenuated forms, skeletons of the ampler and better fed blood organisms, can thus in rod-like relics be detected in the sputa of the tuberculous; while débris of similar micrococci leak into the bullæ of pemphigus and lepra, or drip from the person in the greenish-yellow tears shed by the urethra, which is penitently mourning for its sins in the past.

We understand that the Chicago bacterium is to be cultivated, and in soup! By all means the best of *consommé* for such a genial guest! And let the strain be carefully preserved, with the jealous care, indeed, of those who have now so long guarded the virtues of the "Beaugency" stock.

On such a soup stock as this, one would scarcely dare to set a value, so soon as its real merits were made known to the world at large. In comparison with it, how helpless lies the prescription of that obliging clergyman, whose sands of life have been running out, lo, these many years! Upon how slender a basis, too, rest the claims of that sovereign compound, whose trade-mark, plastered upon every print in the country, are the sedate features of a matronly female, behind whom, it is said, are the faces of two young men in Boston, grinning at the easy credulity of the weaker sex. Indeed, in comparison with such a blood remedy, for which children might well cry and adults lament, do not all others seem inert and destitute of virtue!

Upon what it may be competent to accomplish in other cases, we are not in position now to pronounce, though one might well be dazzled by the brilliancy of the prospect. We can merely speak with confidence of the searching test to which it has been subjected in this city, under circumstances to which attention is here directed. For assuredly it carried one boy safely through a frightful attack of tetanus, and did not desert him when he was long convalescent from the malady; sustained his mother, nursing with sore anxiety her only son, on what she fully believed to be his death-bed; gave her, at the same time, the strength to suckle an infant at the breast, which, at latest accounts, is a noteworthy example of a blooming and round-limbed baby; threw its protecting ægis over still another child of the family, a little girl, who also survives in a vigorous and rosy condition of health, and, lastly, enabled the mother, at the same time, to attend to the numerous calls of her customers, to whom she was in the habit of dispensing over her counters a superior quality of foaming lager beer!

Is there any one, who, under the circumstances, will refuse to assent to the proposition, that this Chicago bacterium, is a "good thing to have in the family?"

---

IT is claimed by one writer, says a contemporary, that one and one-fourth more money is expended annually in funerals in the United States than the government expends for public school purposes, and that funerals cost annually more money than the combined gold and silver yield of the country in the year 1880. This does not include the cost of cemetery lots and burial fees.

ONE hundred and ninety-six bodies have been cremated in Milan in the last five years.

